# Three new species of *Laccaria* (Agaricales, Basidiomycota) from Southwest China (Yunnan) based on morphological and multi-gene sequence data

**DOI:** 10.3389/fmicb.2024.1411488

**Published:** 2024-08-07

**Authors:** Jing Li, Nian-Jie Che, Yang-Yang Cui

**Affiliations:** ^1^Faculty of Geography, Yunnan Normal University, Kunming, China; ^2^CAS Key Laboratory for Plant Diversity and Biogeography of East Asia, Kunming Institute of Botany, Chinese Academy of Sciences (CAS), Kunming, China; ^3^Yunnan Key Laboratory for Fungal Diversity and Green Development, Kunming, China

**Keywords:** morphological characters, multi-gene sequence, novel species, *Laccaria*, taxonomy

## Abstract

**Introduction:**

The genus *Laccaria* has been reported from temperate and tropical areas and is an important constituent in forest ecosystems. However, the species diversity of *Laccaria* in Southwest China (Yunnan) has been underestimated.

**Methods:**

In this paper, descriptions based on morphological and multi-gene sequence data from internal transcribed spacer (ITS) region, large subunit ribosomal RNA gene (nrLSU), translation elongation factor 1-α (*TEF*1α) and the polymerase II second largest subunit (*RPB*2) of three new *Laccaria* species from Southwest China (Yunnan) are reported.

**Results:**

Two of these were characterized by orange pileus and globose to subglobose basidiospores: *L. cinnabarina* and *L. spinulosa*. While *L. cinnabarina* has orange red colored basidiocarps with conspicuously pellucid-striate pattern, and a fibrillose stipe with longitudinally striations, *L. spinulosa* has a brownish orange to brown fruiting body with light white pruinae and 2-spored basidia. *Laccaria longistriata* is characterized by brown to flesh-colored basidioma, prominently striate to sulcate pileus and globose to subglobose basidiospores.

**Discussion:**

The three new species were described, illustrated and compared with closely related species in morphology and phylogeny.

## 1 Introduction

*Laccaria* Berk. & Broome (Hydnangiaceae) is an important ectomycorrhizal (ECM) genus with significant ecological and economic value and a cosmopolitan distribution (Mueller, 1992; Kropp and Mueller, 1999; Wu et al., 2019). Species in this genus, which range from host generalists to host specialists, play a crucial role as symbionts with trees (Mueller, 1992; Roy et al., 2008; Wilson et al., 2017; Herrera et al., 2022). Additionally, *Laccaria* species are known to be pioneers in difficult environments and they are advantageous for both primary and secondary succession in forest ecosystems (Nara et al., 2003). Several *Laccaria* species possess therapeutic properties that are vital for human health while also being edible and tasty (Dai et al., 2009; Wu et al., 2019).

Due to its mostly orange or violet-colored basidioma and echinulate basidiospores, the genus *Laccaria* is morphologically distinct from other genera in the Hydnangiaceae family (Berkeley and Broome, 1883; Mueller, 1984, 1991, 1992; Vellinga, 1986; Wang et al., 2004). This genus has been the focus of several taxonomic studies, mostly in North America or Europe (Heinemann, 1964; Singer, 1967, 1986; Besson and Kühner, 1971; Bon, 1983; Ballero and Contu, 1989; May, 1991; Pázmány, 1994; Kropp and Mueller, 1999; Osmundson et al., 2005; Dovana et al., 2021). However, species delimitation has long been problematic due to phenotypic plasticity and morphological stasis.

In China, approximately 32 *Laccaria* taxa have been identified based on morphological and limited molecular phylogenetic data. These taxa are distributed across more than 20 provinces and regions of China, mostly in Northeast China, East China, South China, and southwest China (Wang et al., 2004; Wilson et al., 2013; Popa et al., 2014; Luo et al., 2016; Vincenot et al., 2017; Corrales et al., 2020; Li, 2020; Cui et al., 2021; Wang et al., 2022; Zhang et al., 2023). However, most of these species bear European and North American names, and their identities are based solely on their morphological and limited molecular characteristics.

The understanding of *Laccaria* species diversity in China remains limited. Therefore, a broader taxon sampling, coupled with both molecular and morphological data, is needed to completely understand the species diversity in China.

In this study, three new species, namely *L. cinnabarina*, *L. longistriata*, and *L. spinulosa*, from Southwest China (Yunnan) have been described with both molecular and morphological data as evidence. These species are generally found in subtropical coniferous and broad-leaved mixed forests.

## 2 Materials and methods

### 2.1 Specimen collection

The 16 specimens examined in this study were collected from Yunnan, China, during the years 2011–2019. They are deposited in the Herbarium of Cryptogams, Kunming Institute of Botany, Chinese Academy of Sciences (KUN-HKAS). Among them, 15 specimens were collected from subtropical broad-leaved forests, while one specimen (KUN-HKAS129615) was collected from a subalpine forest.

### 2.2 DNA extraction, PCR amplification, and DNA sequencing

Genomic DNA was extracted from silica-gel dried or herbarium material using the cetyltrimethylammonium bromide (CTAB) method (Doyle and Doyle, 1987). The universal primer pairs ITS1F/ITS4 (White et al., 1990), LR0R/LR5 (Vilgalys and Hester, 1990), EF1-altertative-3f/EF1-altertative-3r (Stielow et al., 2015), f RPB2-4F/b RPB2-7R, bRPB2-7R2, and bRPB2-7.1R (Liu et al., 1999; Matheny, 2005) were used to amplify the internal transcribed spacer (ITS) region, large subunit ribosomal RNA gene (nrLSU), translation elongation factor 1-α (*TEF*1*α*), and the polymerase II second largest subunit (*RPB*2), respectively. However, we were unable to obtain PCR products from many specimens using *RPB*2 primers. Instead, new primers, LRPB2_F_new (5′-TTWSGWATGCTWTTCCGAAA-3′)/LRPB2_R_new (5′-GGGAAAGGWATWATGCTGGCW-3′), were designed using Primer 3 (version 0.4.0) (Rozen and Skaletsky, 2000) based on sequences available in GenBank and the sequences newly generated in this study.

The PCR reaction was conducted on a SimpliAmp^™^ Thermal Cycler (Applied Biosystems, Foster City, CA, United States) under the following conditions: 94°C for 4 min, followed by 35 cycles of 94°C for 40 s, 52°C for 1 min, and 72°C for 1 min, with a final extension at 72°C for 8 min. The PCR products were purified using a Gel Extraction and PCR Purification Combo Kit (Spin-Column; Bioteke Corporation, Beijing, China). The purified products were sent to Sangon Biotech (Shanghai, China) for sequencing on an ABI-3730-XL sequence analyzer (Applied Biosystems, Foster City, CA, United States) using the same primer combination as for the PCR.

### 2.3 Phylogenetic analyses

Sequences newly generated and retrieved from GenBank are listed in Table 1. *Mythicomyces corneipes* (Fr.) Redhead & A.H. Sm. was chosen as an outgroup according to recent phylogenetic studies by Wilson et al. (2017). The datasets were then aligned using MAFFT v7.130b (Katoh and Standley, 2013) and manually optimized in Bioedit v7.0.9 (Hall, 1999). Phylogenetic trees for multi-loci and single-locus datasets were conducted using maximum likelihood (ML) and Bayesian inference (BI) analyses, which are based on RAxML v. 7.2.6 (Stamatakis, 2006) and MrBayes v. 3.1.2 (Ronquist and Huelsenbeck, 2003), respectively. The most appropriate substitution models for the four datasets were chosen using MrModeltest v. 2.3 (Nylander, 2004) under the Akaike information criterion (AIC).

**Table 1:** Taxa included in molecular phylogenetic analyses and their GenBank accession numbers for ITS, nrLSU, *TEF*1*α*, and *RPB*2 sequences.

Species	Voucher	Locality	GenBank accession numbers				References
			ITS	nrLSU	TEF1α	RPB2	
*Laccaria acanthospora*	AWW485(T)	Tibet, China	JX504102	JX504186	KU686073	KU685916	Wilson et al. (2013)
*L. acanthospora*	KUN-HKAS46089	Tibet, China	JX504162	—	—	—	Wilson et al. (2013)
*L. alba*	F1121461	Tibet, China	JX504129	JX504209	—	—	Wilson et al. (2013)
*L. alba*	ASIS18039	Korea	MG519546	—	MG551652	MG551620	Cho et al. (2018)
*L. amethysteo-occidentalis*	TENN42526(T)	Canada	DQ149848	—	—	—	Wilson et al. (2013)
*L. amethysteo-occidentalis*	DAVFP 28205	Canada	HQ650762	—	—	—	Kranabetter et al. (2012)
*L. amethystina*	GMM7633	France	JX504154	JX504228	—	—	Wilson et al. (2013)
*L. amethystina*	RK01-03	Denmark	AM113955	—	—	—	Sheedy et al. (2013)
*L. angustilamella*	KUN-HKAS58714	Yunnan, China	JX504168	JX504244	—	—	Wilson et al. (2013)
*L. angustilamella*	GMM6171	Yunnan, China	JX504132	—	—	—	Wilson et al. (2013)
*L. angustilamella*	BAP226	Yunnan, China	JX504118	JX504201	—	—	Wilson et al. (2013)
*L. anthracina*	HMAS254678(T)	Tibet, China	KX496973	—	—	—	Wang et al. (2022)
*L. anthracina*	HMAS260494	Tibet, China	KX496974	—	—	—	Wang et al. (2022)
*L. anthracina*	HMAS274263	Tibet, China	KX496975	—	—	—	Wang et al. (2022)
*L. araneosa*	KNU20120912-40(T)	Korea	MG519548	MG519588	MG551654	MG551621	Cho et al. (2020)
*L. araneosa*	KNU20120912-25	Korea	MG519550	MG519590	MG551656	MG551623	Cho et al. (2018)
*L. araneosa*	HMAS97019	Jilin, China	ON877218	—	—	—	Wang et al. (2022)
*L. aurantia*	MB-FB-001106(T)	Yunnan, China	JQ670895	—	—	—	Popa et al. (2014)
*L. aurantia*	MB-FB-001109	Yunnan, China	JQ681209	—	—	—	Popa et al. (2014)
*L. bicolor*	S238N	Sweden	DQ179123	—	—	—	Wilson et al. (2017)
*L. bicolor*	GMM2118	USA	KU685635	—	—	—	Wilson et al. (2017)
*L. bullipellis*	AWW465(T)	Tibet, China	JX504100	JX504184	—	KU685914	Wilson et al. (2017)
*L. canaliculata*	GMM7267	Australia	JX504137	JX504213	KU686093	KU685960	Wilson et al. (2017)
*L. canaliculata*	GMM7251	Australia	KU685669	KU685812	KU686090	KU685955	Wilson et al. (2017)
*L. cinnabarina*	**KUN-HKAS83381**	**Yunnan, China**	**OR722588**	**OR722601**	**PP171545**	**PP171558**	**This study**
*L. cinnabarina*	**KUN-HKAS74787**	**Yunnan, China**	**OR722586**	**OR722599**	**PP171546**	**PP171556**	**This study**
*L. cinnabarina*	**KUN-HKAS79704**	**Yunnan, China**	**OR722589**	**OR722600**	**PP171547**	**PP171557**	**This study**
*L. cinnabarina*	**KUN-HKAS80885(H)**	**Yunnan, China**	**OR722587**	**OR722595**	—	—	**This study**
*L. darjeelingensis*	CUHAM788(T)	India	OQ607624	—	—	—	Thapa et al. (2024)
*L. fagacicola*	KUN-HKAS90435(H)	Yunnan, China	MW540806	**OR722593**	**PP171549**	—	Cui et al. (2021), **this study**
*L. fagacicola*	KUN-HKAS107731	Yunnan, China	MW540807	**OR722594**	**PP171550**	**PP171554**	Cui et al. (2021), **this study**
*L. fengkaiensis*	KUN-HKAS106739(H)	Guangdong, China	MN585657	—	—	—	Li (2020)
*L. fengkaiensis*	KUN-HKAS106741	Guangdong, China	MN585658	—	—	—	Li (2020)
*L. fulvogrisea*	MB-FB-001101	Yunnan, China	JQ670896	—	—	—	Popa et al. (2014)
*L. fulvogrisea*	MB-FB-001110	Yunnan, China	JQ681210	—	—	—	Popa et al. (2014)
*L. galerinoides*	F1081213	Chile	KU685634	KU685778	KU686078	KU685929	Wilson et al. (2017)
*L. galerinoides*	F1080983	Argentina	KU685632	KU685776	KU686077	KU685927	Wilson et al. (2017)
*L. glabripes*	GMM7521	New Zealand	KU685708	KU685849	KU686117	KU685991	Wilson et al. (2017)
*L. glabripes*	GMM7534	New Zealand	KU685711	KU685852	—	—	Wilson et al. (2017)
*L. himalayensis*	AWW484(T)	Tibet, China	JX504101	JX504185	—	KU685915	Wilson et al. (2017)
*L. himalayensis*	AWW495	Tibet, China	JX504104	JX504188	—	—	Wilson et al. (2013)
*L. infundibuliformis*	CUHAM786	India	OQ607560	—	—	—	Thapa et al. (2024)
*L. japonica*	TNS-F64167(T)	Japan	KU962988	—	—	—	Vincenot et al. (2017)
*L. japonica*	SFC20110921-34	Korea	MG519519	MG519568	—	MG551596	Cho et al. (2018)
*L. laccata*	GMM7615	France	JX504148	JX504222	—	—	Wilson et al. (2013)
*L. laccata* var. *pallidifolia*	GMM7605	France	JX504146	—	—	—	Wang et al. (2022)
*L. laccata* var. *pallidifolia*	HMAS293215	Hebei, China	ON877219	—	—	—	Wang et al. (2022)
*L. laccata* var. *pallidifolia*	CLC1724(MONT)	USA	DQ149857	—	—	—	Wang et al. (2022)
*L. lateritia*	RGB 166658	Malaysia	JN235950	—	—	—	Luo et al. (2016)
*L. lateritia*	RGB 166659	Malaysia	JN235949	—	—	—	Luo et al. (2016)
***L. longistriata***	**KUN-HKAS123799**	**Yunnan, China**	**OQ396727**	**OR345239**	**OR347684**	**OR347686**	**This study**
***L. longistriata***	**KUN-HKAS123800**	**Yunnan, China**	**OQ396728**	**OR345240**	**OR347683**	**OR347687**	**This study**
***L. longistriata***	**KUN-HKAS123801(H)**	**Yunnan, China**	**OQ396730**	—	**OR347685**	—	**This study**
***L. longistriata***	**KUN-HKAS115989**	**Yunnan, China**	**OQ396729**	—	**OR347682**	**OR347688**	**This study**
*L. macrocystidia*	GMM7616	France	KM067850	KU685863	—	KU686004	Wilson et al. (2017)
*L. macrocystidia*	GMM7612	France	KM067847	KU685861	KU686122	KU686002	Wilson et al. (2017)
*L. macrocystidiata*	GDOR5080(E)	Italy	MW584890	—	—	—	Dovana et al. (2021)
*L. macrocystidiata*	GDOR5079	Italy	MW584891	—	—	—	Dovana et al. (2021)
*L. macrocystidiata*	GDOR5075	Greece	MW584893	—	—	—	Dovana et al. (2021)
*L. miniata*	GDGM76043(T)	Guangdong, China	OR689440	OR785476	—	—	Zhang et al. (2023)
*L. montana*	TWO591(MONT)	—	DQ149865	—	—	—	Osmundson et al. (2005)
*L. montana*	TWO319(MONT)	—	DQ149862	—	—	—	Osmundson et al. (2005)
“*L. montana*”	TENN42882	—	DQ149860	—	—	—	Osmundson et al. (2005)
*L. moshuijun*	KUN-HKAS93732(H)	Yunnan, China	KU962989	—	—	—	Vincenot et al. (2017)
*L. moshuijun*	HMAS131870	Yunnan, China	ON877154	—	—	—	Wang et al. (2022)
*L. moshuijun*	HMAS264430	Yunnan, China	ON877171	—	—	—	Wang et al. (2022)
*L. murina*	ASIS2021	Korea	MG519554	—	—	—	Cho et al. (2020)
*L. murina*	ASIS216	Korea	MG519553	—	—	—	Cho et al. (2020)
*L. nanlingensis*	GDGM84954(T)	Guangdong, China	OR689442	OR785478	OR826273	OR835199	Zhang et al. (2023)
*L. nanlingensis*	GDGM84949	Guangdong, China	OR689441	OR785477	OR826274	OR835198	Zhang et al. (2023)
*L. negrimarginata*	BAP360(T)	Tibet, China	JX504120	—	—	—	Wilson et al. (2013)
*L. negrimarginata*	GMM7631	Tibet, China	JX504153	JX504227	KU686130	KU686011	Wilson et al. (2017)
*L. neovinaceoavellanea*	GDGM52852(T)	Jiangxi, China	OR689447	OR785479	—	—	Zhang et al. (2023)
*L. neovinaceoavellanea*	GDGM53063	Jiangxi, China	OR689448	OR785480	—	—	Zhang et al. (2023)
*L. neovinaceoavellanea*	GDGM89621	Yunnan, China	OR689449	OR785481	—	—	Zhang et al. (2023)
*L. ohiensis*	GMM7564	New Zealand	KU685715	KU685856	—	KU685997	Wilson et al. (2013, 2017)
*L. pallidorosea*	KUN-HKAS107730(H)	Yunnan, China	MW540808	—	—	—	Cui et al. (2021), **This study**
*L. pallidorosea*	KUN-HKAS53170	Yunnan, China	MW540809	**OR722602**	**PP171548**	**PP171555**	Cui et al. (2021), **This study**
*L. pallidus*	CUHAM787	India	OQ607623	—	—	—	Thapa et al. (2024)
*L. paraphysata*	PDD:80007	New Zealand	KM975424	—	—	—	Direct Submission
*L. paraphysata*	PDD:95230	New Zealand	KM975427	—	—	—	Direct Submission
*L. parva*	SFC20120919-40(T)	Korea	MG519525	—	—	MG551600	Cho et al. (2020)
*L. parva*	KUN-HKAS107732	Yunnan, China	MW540810	—	—	—	Cui et al. (2021)
*L. prava*	KUN-HKAS106745	Guangdong, China	MN585661	—	—	—	Li (2020)
*L. prava*	KUN-HKAS106742(H)	Guangdong, China	MN585660	—	—	—	Li (2020)
*L. pumila*	GMM7637	France	JX504156	JX504229	KU686158	—	Wilson et al. (2017)
*L. pumila*	HMAS293222	Hebei, China	ON877220	—	—	—	Wang et al. (2022)
*L. rubroalba*	KUN-HKAS90766	Yunnan, China	KX449359	—	—	—	Luo et al. (2016)
*L. rubroalba*	KUN-HKAS90751	Yunnan, China	KX449360	—	—	—	Luo et al. (2016)
*L. rubroalba*	KUN-HKAS90753(H)	Yunnan, China	KX449358	—	—	—	Luo et al. (2016)
*L. rufobrunnea*	GDGM82878(T)	Yunnan, China	OR689443	OR785482	OR826272	OR835197	Zhang et al. (2023)
*L. rufobrunnea*	GDGM89627	Yunnan, China	OR689444	OR785483	—	—	Zhang et al. (2023)
*L. salmonicolor*	GMM7596	Tibet, China	JX504143	JX504218	KU686151	KU686045	Wilson et al. (2017)
*L. salmonicolor*	GMM7602(T)	Tibet, China	JX504145	JX504220	—	—	Wilson et al. (2013)
***L. spinulosa***	**KUN-HKAS122272**	**Yunnan, China**	**OR722592**	**OR722596**	**PP171552**	—	**This study**
***L. spinulosa***	**KUN-HKAS90444**	**Yunnan, China**	**OR722590**	**OR722597**	**PP171553**	—	**This study**
***L. spinulosa***	**KUN-HKAS129615(H)**	**Yunnan, China**	**OR722591**	**OR722598**	**PP171551**	—	**This study**
*L. stellata*	Corrales27	Panama	MT279231	MT279210	—	MT431185	Corrales et al. (2020)
*L. stellata*	SYC109	Panama	KP877340	—	—	—	Popa et al. (2016)
*L. stellata*	SYC207	Panama	KP877339	—	—	—	Popa et al. (2016)
*L. striatula*	TENN070507	USA	KY777385	—	—	—	Direct Submission
*L. striatula*	NAMA2017-345	USA	MH979278	—	—	—	Direct Submission
*L. striatula*	iNAT99997025	USA	ON206675	—	—	—	Direct Submission
*L. trichodermophora*	GMM7733	USA	JX504157	JX504230	—	KU686013	Wilson et al. (2017)
*L. trichodermophora*	KH_LA06_012	USA	KM067880	—	—	—	Wilson et al. (2017)
*L. trichodermophora*	KH_LA06_013	USA	KM067881	—	—	—	Wilson et al. (2017)
*L. tortilis*	ASIS22273	Korea	MG519533	MG519576	MG551644	MG551608	Cho et al. (2020)
*L. tortilis*	GMM7635	France	KM067859	KU685906	KU686156	KU686053	Wilson et al. (2017)
*L. umbilicata*	GDGM82911(T)	Yunnan, China	OR689446	OR785486	OR826268	OR835192	Zhang et al. (2023)
*L. umbilicata*	GDGM82883	Yunnan, China	OR689445	OR785485	OR826270	OR835194	Zhang et al. (2023)
*L. versiforma*	SFC20120926-01	Korea	MG519556	MG519594	MG551660	MG551627	Cho et al. (2020)
*L. vinaceoavellanea*	SFC20120922-02	Korea	MG519535	—	—	MG551610	Cho et al. (2020)
*L. yunnanensis*	MB-FB-001107(T)	Yunnan, China	JQ670897	—	—	—	Popa et al. (2014)
*L. yunnanensis*	MB-FB-001108	Yunnan, China	JQ681208	—	—	—	Popa et al. (2014)
“*Laccaria* sp.”	A0561	Japan	JX504082	—	—	—	Wilson et al. (2017)
“*Laccaria* sp.”	A0577	Japan	KU685619	—	—	—	Wilson et al. (2017)
“*Laccaria* sp.”	A0087	Japan	KU685614	—	—	—	Wilson et al. (2017)
“*Laccaria* sp.”	A0151	Japan	JX504081	—	—	—	Wilson et al. (2013)
“*Laccaria* sp.”	A2861	Japan	JX504086	—	—	—	Wilson et al. (2013)
“*Laccaria* sp.”	AWW593	USA	JX504113	JX504196	—	—	Wilson et al. (2017)
“*Laccaria* sp.”	SB2135	Portugal	JX504172	JX504249	KU686140	KU686028	Wilson et al. (2017)
*Mythicomyces corneipes*	AFTOL972	Germany	DQ404393	—	DQ029197	DQ408110	Sheedy et al. (2013)
*M. corneipes*	ES11.10.2.A	Germany	KC964108	—	—	—	Luo et al. (2016)

Accession numbers in bold font indicate a newly generated sequence in this study.

Statistical support for the phylogenetic trees was calculated using non-parametric bootstrapping with 1,000 replicates for ML analysis (ML bootstrapping: MLB). For BI analyses, the selected models and four chains were used, and the analysis was stopped when the standard deviation of the split frequencies fell below 0.01 and the effective sample size (ESS) values were >200. Tracer v 1.7 (Rambaut et al., 2018) was used to monitor chain convergence. Trees were sampled every 100 generations. Subsequently, the trees were summarized, and statistical support was obtained using the “sump” and “sumt” commands in MrBayes by discarding the first 25% of generations as burn-ins. Bayesian posterior probabilities (BPP) for internodes were estimated based on the majority rule consensus with the remaining trees.

### 2.4 Morphological studies

Field notes and digital photos were used to describe macroscopic features, with color codes based on the study by Kornerup and Wanscher (1981). Microscopic characteristics were observed on dried specimens mounted in 5% KOH and stained with Congo red when necessary, under a microscope. The notation “[*n*/*m*/*p*]” denotes the measurement of *n* basidiospores from *m* basidiomata of *p* collections. The size of basidiospores is represented as “(*a*–) *b*–*c* (−*d*),” where at least 90% of the measured values fall within the range *b*–*c*.

Parentheses are used to indicate extreme values in a and d. The symbol $\bar{x}$ represents the average length of basidiospores ± sample standard deviation × average width of basidiospores ± sample standard deviation. *Q* is the length-to-width ratio of a basidiospore in the side view. *Q*_m_ indicates the average *Q* of all measured basidiospores ± sample standard deviation. SigmaPlot 10.0 was used to calculate these measurements (Systat Software, San Jose, CA).

With a phase contrast objective (× 1,000), line drawings of the new species were illustrated by placing a hand under the microscope. The scanning electron microscopy (SEM) was applied to observe the characters of basidiospores.

## 3 Results

### 3.1 Phylogenetic analyses

In total, 44 sequences generated in this study were included in the single-locus and multi-loci datasets. For the four single-locus datasets, the GTR + GAMMA + I model was identified as the most appropriate substitution model for ITS, nrLSU, and *RPB*2, while the SYM + GAMMA + I model was found to be the most suitable one for *TEF*1*α*. The aligned four-gene matrix contained a total of 126 samples, with 3,693 aligned bases per sample.

**Figure 1:**
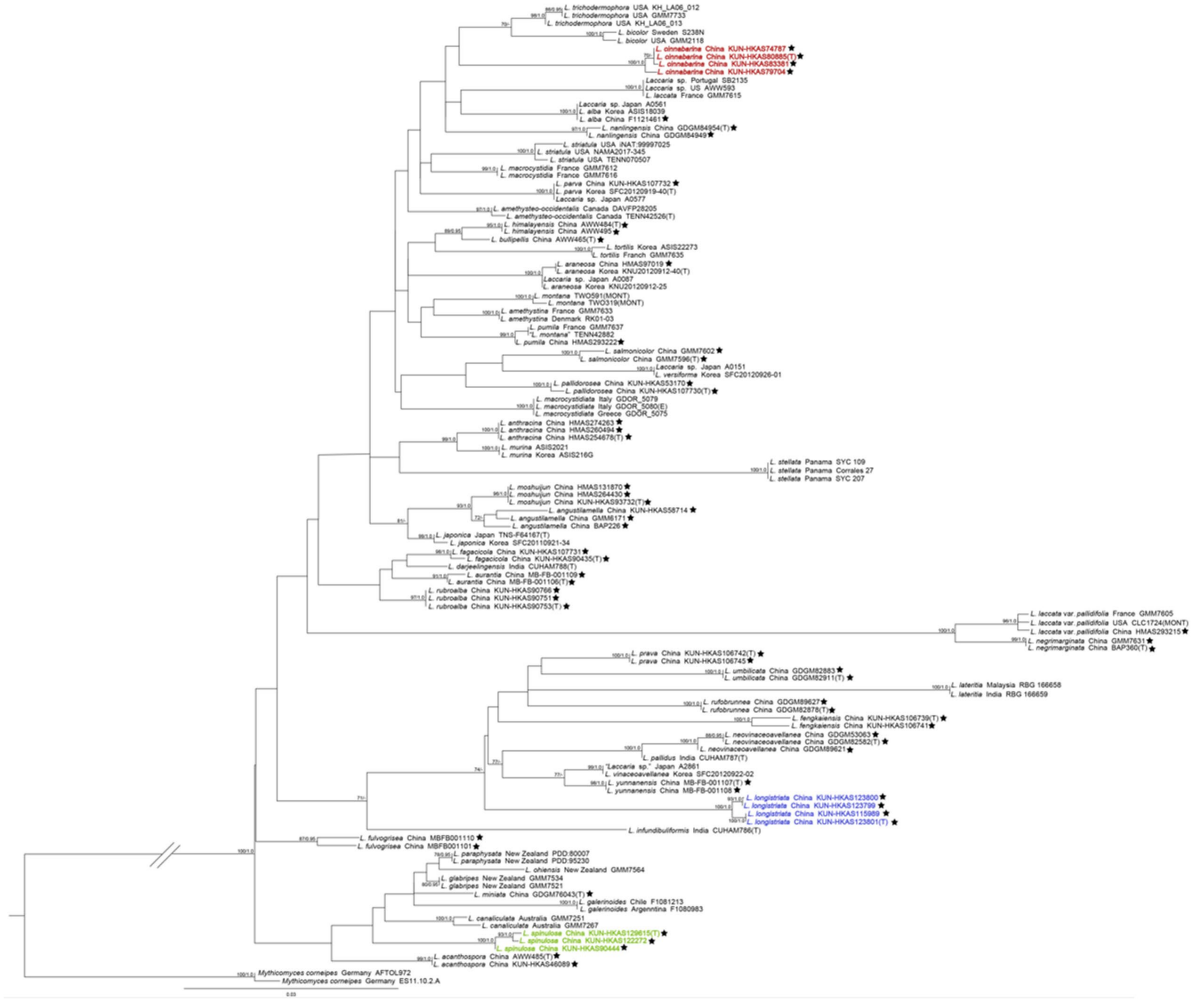
The maximum likelihood (ML) tree of *Laccaria* was inferred from the multi-locus dataset (ITS, nrLSU, *TEF*1*α*, and *RPB*2). ML bootstrap values over 70% (MLB ≥70%) and Bayesian posterior probabilities over 0.90 (BPP ≥0.90) are shown above or beneath individual branches. Sequences from type, holotype, or epitype specimens are marked with (T), (H), or (E), respectively; the three new species are indicated in red, purple, and green boldface, and species identified in China are labelled with a black pentagram. The double slash was used to optimize the phylogenetic tree, which has a long branch between *Laccaria* and *Mythicomyces*.

The phylogenetic trees of the *Laccaria* species complex were constructed based on these single-locus and four-gene matrices. Taking into consideration that the topology of the phylogenetic trees based on the four-gene dataset generated from ML and BI analyses was almost identical, with only slight differences in statistical support, only the tree inferred from the ML analysis is displayed (Figure 1).

In our multi-loci phylogenetic analysis, the three new species occupied independent positions in *Laccaria*, each with high support (MLB/BPP = 100%/1.0). *Laccaria cinnabarina* is related to *Laccaria trichodermophora* G. M. Mueller and *Laccaria bicolor* (Maire) P. D. Orton. *Laccaria longistriata* clustered together with *Laccaria yunnanensis* Popa et al., *Laccaria vinaceoavellanea* Hongo, and *Laccaria pallidus* A. Thapa and K. Acharya, *Laccaria neovinaceoavellanea* Ming Zhang and X. L. Gao, *Laccaria fengkaiensis* Fang Li, *Laccaria rufobrunnea* Ming Zhang and X. L. Gao, *Laccaria lateritia* Malençon, *Laccaria umblilicata* Ming Zhang, and *Laccaria prava* Fang Li. Three specimens of *L. spinulosa* formed a monophyletic clade close to *Laccaria acanthospora* A. W. Wilson and G. M. Mueller, *Laccaria canaliculata* (Sacc.) Massee, *Laccaria galerinoides* Singer, *Laccaria miniata* Ming Zhang, *Laccaria glabripes* McNabb, *Laccaria ohiensis* (Mont.) Singer, and *Laccaria paraphysata* (McNabb) J. A. Cooper at the base of the genus.

### 3.2 Morphological observations

**Figure 2:**
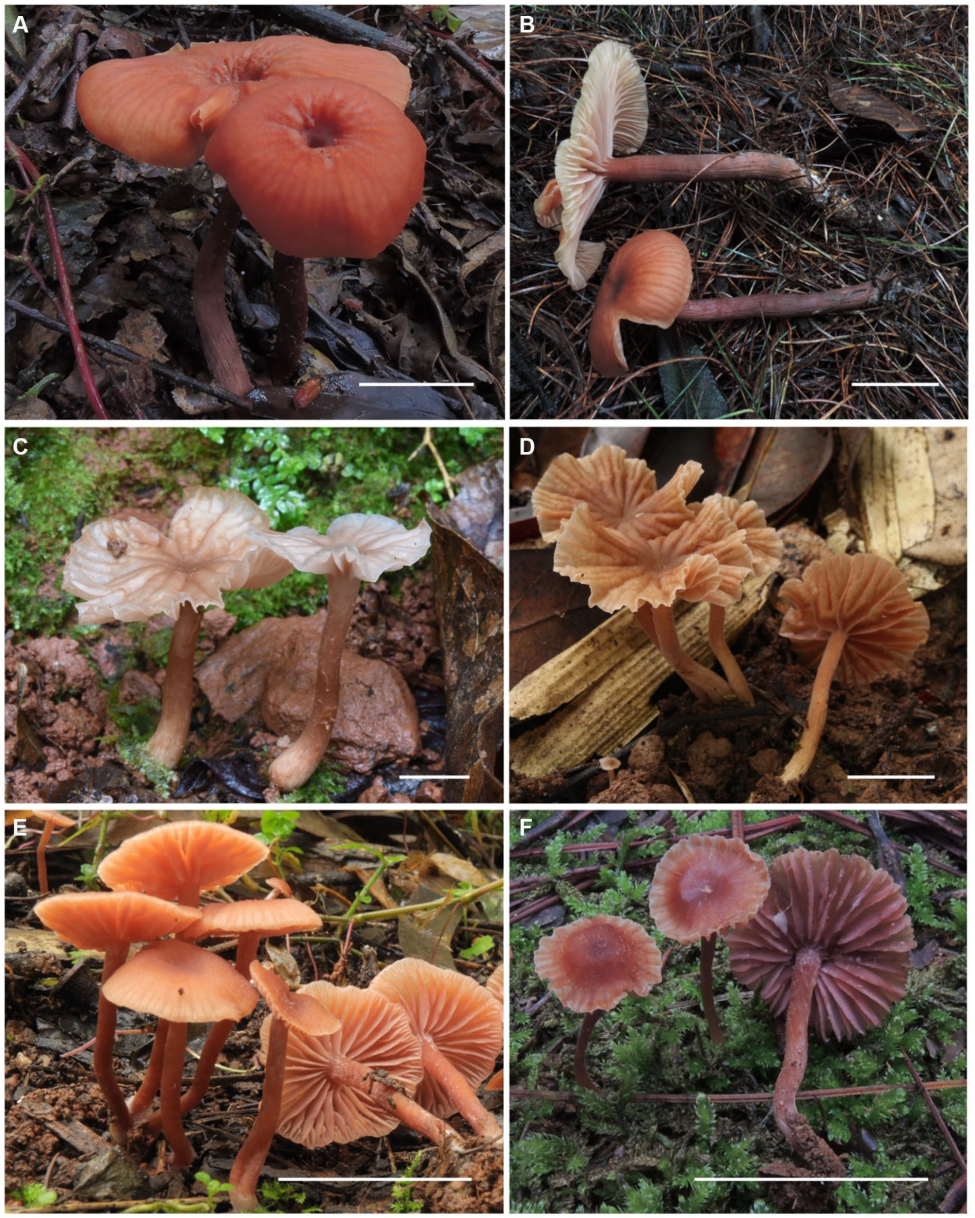
Fresh basidiomata of *Laccaria cinnabarina*, *Laccaria longistriata*, and *Laccaria spinulosa*. **(A,B)** *Laccaria cinnabarina* (a holotype, KUN-HKAS80885, b KUN-HKAS83302). **(C,D)** *Laccaria longistriata* (**C** holotype, KUN-HKAS123801, **D** KUN-HKAS123317). **(E,F)** *Laccaria spinulosa* (**E** holotype, KUN-HKAS129615, **F** KUN-HKAS90444). Bars: 2 cm.

**Figure 3:**
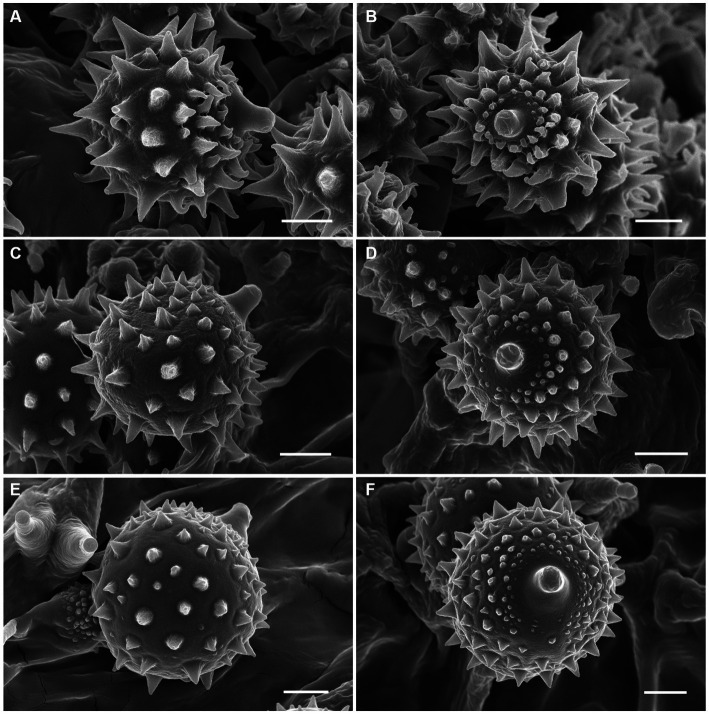
Basidiospores of *Laccaria cinnabarina*, *Laccaria longistriata*, and *Laccaria spinulosa* under SEM. **(A,B)** *Laccaria cinnabarina* (holotype, KUN-HKAS80885). **(C,D)** *Laccaria longistriata* (holotype, KUN-HKAS123801). **(E,F)** *Laccaria spinulosa* (holotype, KUN-HKAS129615). Bars: 2 μm.

**Figure 4:**
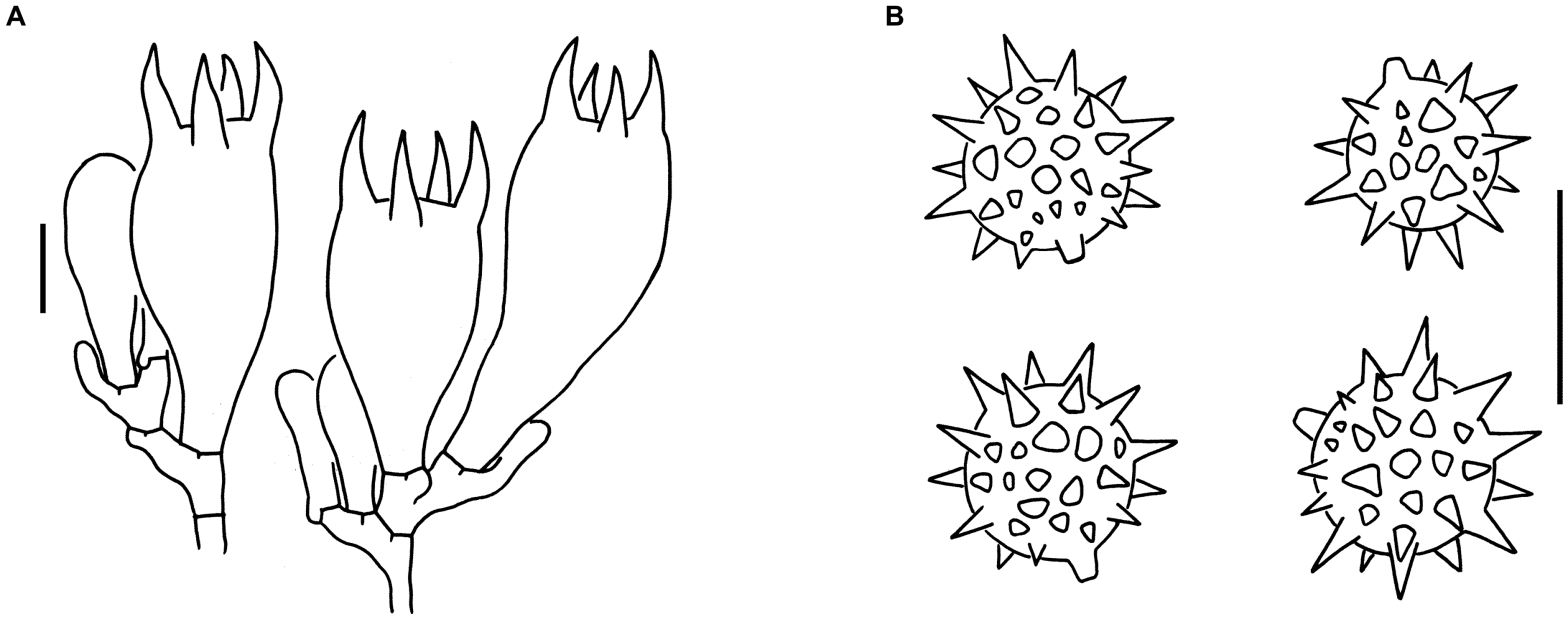
Microscopic features of *Laccaria cinnabarina* (holotype, KUN-HKAS80885). **(A)** Hymenium and subhymenium. **(B)** Basidiospores. Bars: **A,B** = 10 μm.

**Figure 5:**
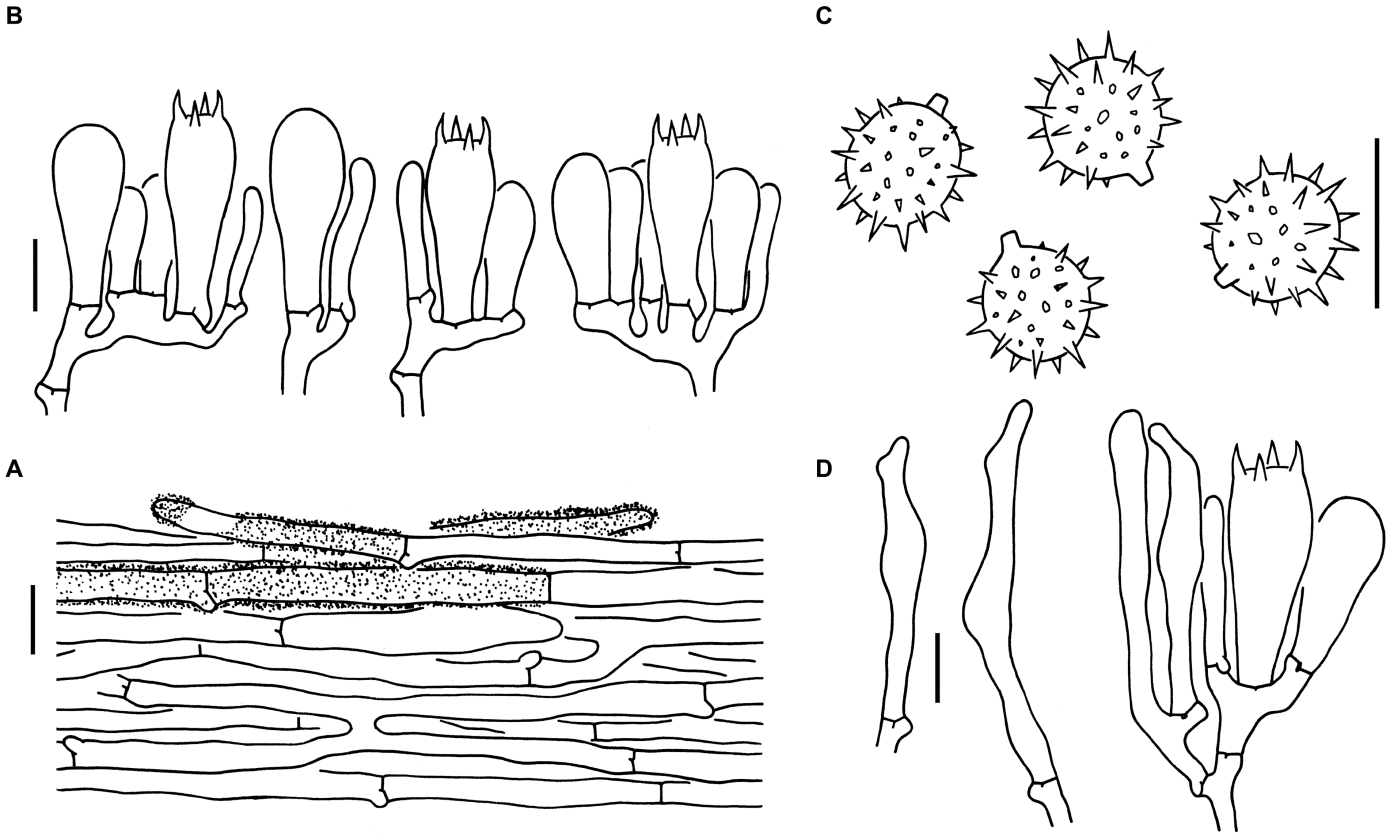
Microscopic features of *Laccaria longistriata* (holotype, KUN-HKAS123801). **(A)** Pileipellis. **(B)** Hymenium and subhymenium. **(C)** Basidiospores. **(D)** Marginal cells in the lamellar edge. Bars: **A** = 20 μm, **B–D** = 10 μm.

**Figure 6:**
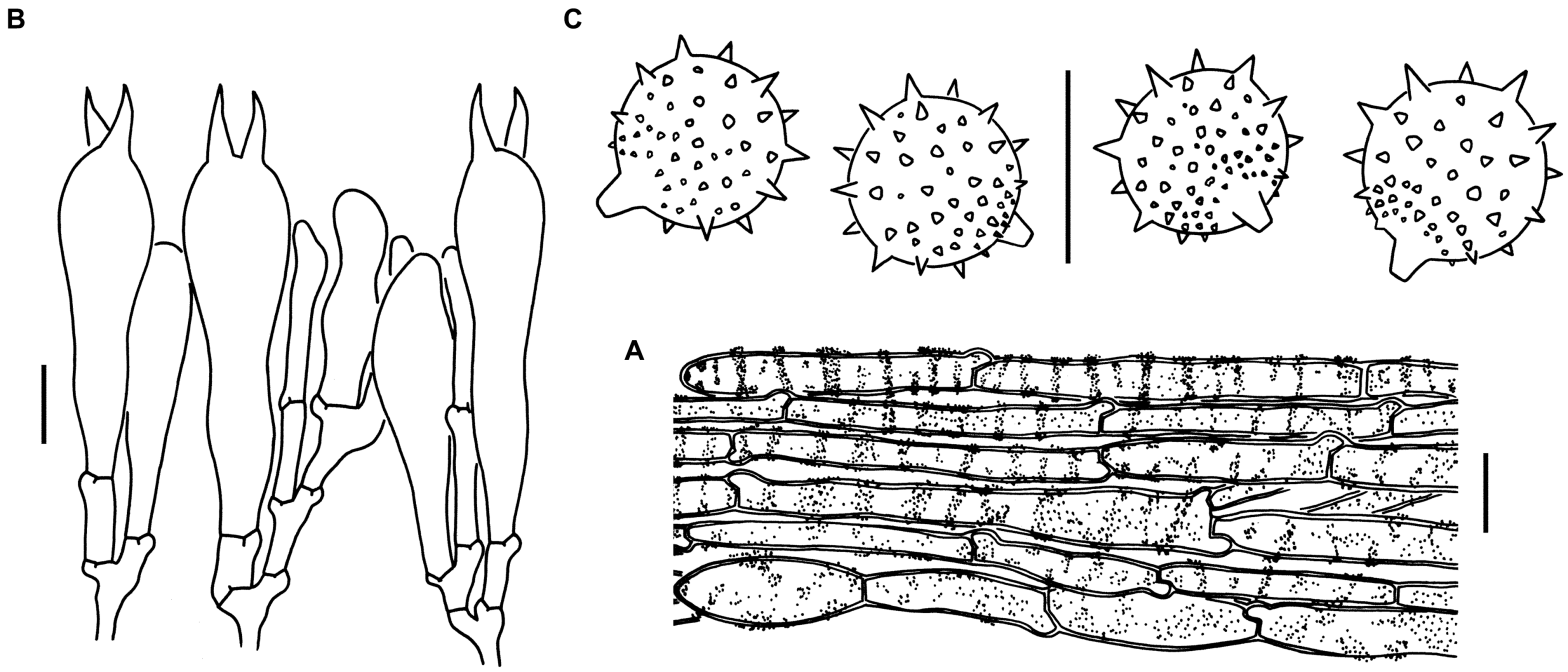
Microscopic features of *Laccaria spinulosa* (holotype, KUN-HKAS129615). **(A)** Pileipellis. **(B)** Hymenium and subhymenium. **(C)** Basidiospores. Bars: **A** = 20 μm, **B,C** = 10 μm.

Sixteen specimens representing three new species (*L. cinnabarina*, *L. longistriata*, and *L. spinulosa*) were morphologically examined (Figure 2). The SEM images of basidiospores are presented in Figure 3. The line drawings of the three new species from the type specimen are provided in Figures 4–6.

### 3.3 Taxonomy

***Laccaria cinnabarina*** J. Li and Y. Y. Cui, sp. nov.

Figures 2A,B, 3A,B, 4.

**MycoBank No.**: MB 850637.

**Etymology**: The species name *cinnabarina* refers to its orange-red pileus.

**Diagnosis**: Similar to *Laccaria himalayensis* A. W. Wilson and G. M. Mueller but differs by its very small to medium-sized pileus, dark reddish-brown stipe, and globose to subglobose basidiospores.

**Holotype**: China. Yunnan Province: Lvchun County, 22°30′18″ N, 102°03′59″ E, altitude 2,300 m, in a broad-leaved forest with trees of Fagaceae, 26 May 2013, Qi Zhao 1778 (KUN-HKAS80885).

**Description**:

- **Basidioma**: Very small to medium-sized.
- **Pileus**: 1–9 cm in diameter; convex to applanate, with an obvious depressed at the center; dark brown (6E6–8) at the center and orange-red (6B6–8) toward the margin; conspicuously pellucid-striate, slightly sulcate; context cream (1A2) to orange (6A2–5).
- **Lamellae**: Sinuate to adnate; slightly distant, sometimes furcate and intervenose at the margin; pinkish (5A2–4) to pale orange (6A2); lamellulae attenuate.
- **Stipe**: 3–9 × 0.2–1 cm, cylindrical, fibrillose, longitudinally striate, dark reddish brown (6E4–8); basal mycelium white (1A1).
- **Pileipellis**: Composed of ± radially arranged, slightly thick-walled (ca. 0.5 μm), orange-yellow filamentous hyphae that are 2–9 μm wide.
- **Basidia**: 30–45 × 14–16 μm, clavate, hyaline, 4-spored; sterigmata 6–10 μm long.
- **Basidiospores**: (Excluding ornamentation) [100/5/5] (6.5–) 7–9.5 (−10.5) × (6–) 7–9 (−9.5) μm, $\bar{x}$ = 8.3 ± 0.7 × 8 ± 0.7, *Q* = 1–1.11 (−1.25), *Q*_m_ = 1.05 ± 0.04, globose to subglobose, hyaline, and echinate; spines ca. 2 μm long, up to 2 μm wide at the base and crowded; hilar appendix that is 1.0–1.5 μm long and prominent.
- **Pleurocystidia and cheilocystidia**: Lacking.
- **Subhymenium**: Composed of filamentous hyphae that are 2–3 μm wide.
- **Lamellar trama**: Regular, composed of a slightly thick-walled (ca. 0.5 μm) filamentous hyphae that are 6–12 μm wide.
- **Stipitipellis**: Composed of appressed, parallel, simply septate, thin to slightly thick-walled (ca. 0.5 μm), colorless to pale yellow hyphae that are 2–11 μm wide.
- **Caulocystidia**: Lacking.
- **Clamps**: Present in all parts of the basidioma.

**Habitat and distribution**: Found singly, scattered, or in groups on soil in subtropical broad-leaved forests with trees of Fagaceae. Basidioma occurs from summer to autumn. It is known from southwest China (Yunnan).

Additional specimens studied: China

Yunnan Province:

- Longling County, 24°07′52″ N, 98°25′46″ E, altitude 2,500 m, in a coniferous and broad-leaved mixed forest, 16 June 2014, Jiao Qin 885 (KUN-HKAS83302)
- Longling County, altitude 2,480 m, in a coniferous and broad-leaved mixed forest, 29 July 2014, Jiao Qin 964 (KUN-HKAS83381)
- Fugong County, 26°54′06″ N, 98°52′40″ E, altitude 1,370 m, in a coniferous and broad-leaved mixed forest, 2 August 2011, Gang Wu 476 (KUN-HKAS74787)
- Jingdong Yi Autonomous County, 24°23′06″ N, 100°50′44″ E, altitude 2,491 m, in a coniferous and broad-leaved mixed forest, 22 July 2013, Yang-Yang Cui 24 (KUN-HKAS79704).

***Laccaria longistriata*** J. Li and Y.Y. Cui, sp. nov.

Figures 2C,D, 3C,D, 5.

**MycoBank No.**: MB 847530.

**Etymology**: The species *longistriata* refers to its conspicuous striations on the pileus.

**Diagnosis**: Similar to *Laccaria yunnanensis* but differs by its very small to small basidioma, slightly larger basidiospores, and occurrence in the Ailao Shan Mountains in Yunnan at an altitude of approximately 2,500 m.

**Holotype**: China. Yunnan Province: Mengla County, 21°45′52″ N, 101°56′46″ E, altitude 960 m, in a broad-leaved forest with trees of Fagaceae, 27 August 2019, Liu-Kun Jia 481 (KUN-HKAS123801).

**Description**:

- **Basidioma**: Very small to small.
- **Pileus**: 0.5–4.5 cm in diameter, plano-convex to applanate and slightly depressed at the center; brownish (6C4–7) to brown (6D5–7) at the center and cream (1A2), flesh-colored (7A5) to brownish (6C4–7) toward the margin; conspicuously striate, often sulcate; context flesh-colored (7A5) to brownish (6C6–8).
- **Lamellae**: Subdecurrent, obviously distant; flesh-colored (7A5), brownish (6C6–8) to brown (6D5–7); lamellulae attenuate.
- **Stipe**: 4–6 × 0.3–0.8 cm, cylindrical, glabrous, brownish (6C3–6) to brown (6D5–7); basal mycelium white (1A1).
- **Pileipellis**: Composed of ± radially arranged, slightly thin-walled (ca. 0.5 μm), colorless to brownish filamentous hyphae that are 4–12 μm wide.
- **Basidia:** 27–42 × 10–13 μm, clavate, hyaline, 4-spored; sterigmata 4–6 μm long.
- **Basidiospores**: (Excluding ornamentation) [90/5/5] 6.5–8 × 6–8 μm, $\bar{x}$= 7.3 ± 0.4 × 7 ± 0.4, *Q* = 1–1.07 (−1.17), *Q*_m_ = 1.02 ± 0.04, globose to subglobose, and hyaline, echinate; spines 0.5–2 μm long, ca. 0.5 μm wide at the base and crowded; hilar appendix ca. 1 μm long and prominent.
- **Pleurocystidia**: Lacking.
- **Marginal cells in lamellar edge**: Fertile.
- **Cheilocystidia**: Present, 30–60 × 4–8 μm, filamentous, narrowly clavate to subcapitate, thin-walled, colorless, hyaline.
- **Subhymenium**: Composed of filamentous hyphae that are 2–5 μm wide. Lamellar trama regular, composed of thin-walled filamentous hyphae that are 2–7 μm wide.
- **Stipitipellis**: Composed of appressed, parallel, simply septate, thin-walled, colorless to brownish hyphae that are 4–7 μm wide.
- **Caulocystidia** 30–35 × 6–12 μm, clavate, slightly thick-walled (up to 1 μm), colorless, clustered in small groups.
- **Clamps**: Present in all parts of basidioma.

**Habitat and distribution**: Found singly, scattered, or in groups on soil in subtropical broad-leaved forests with trees of Fagaceae. Basidioma occurs from summer to autumn. Known from southwest China (Yunnan).

Additional specimens studied: China:

Yunnan Province:

- Mengla County, 21°45′52″ N, 101°56′46″ E, altitude 960 m, in a broad-leaved forest with trees of Fagaceae, 27 August 2019, Geng-Shen Wang 761 (KUN-HKAS121527).
- Same county, altitude 1,000 m, in a broad-leaved forest with trees of Fagaceae, 29 August 2019, Liu-Kun Jia 547 (KUN-HKAS127112).
- Jinghong, 22°01′00″ N, 100°46′17″ E, altitude 1,200 m, in a broad-leaved forest with trees of Fagaceae, 22 August 2019, Geng-Shen Wang 684 (KUN-HKAS123317).
- Same city, altitude 820 m, in a broad-leaved forest with trees of Fagaceae, 21 August 2019, Liu-Kun Jia 349 (KUN-HKAS123800).
- Jiangcheng Hani-Yi Autonomous County, 22°35′06″ N, 101°51′44″ E, altitude 1,000 m, in a broad-leaved forest with trees of Fagaceae, 25 June 2019, Geng-Shen Wang 243 (KUN-HKAS109631).
- Same county, altitude 1,100 m, in a broad-leaved forest with trees of Fagaceae, 28 June 2019, Geng-Shen Wang 288 (KUN-HKAS115989).
- Same county, altitude 1,101 m, in a broad-leaved forest with trees of Fagaceae, 21 September 2019, Sai Gong 912 (KUN-HKAS123799).

***Laccaria spinulosa*** J. Li and Y. Y. Cui, sp. nov.

Figures 2E,F, 3E,F, 6.

**MycoBank No.**: MB 850641.

**Etymology**: The species name *spinulosa* refers to its spinulose basidiospores.

**Diagnosis**: Similar to *Laccaria pumila* Fayod but differs by its pinkish flesh lamellae, pileus, and stipe, both with light white pruinae, and smaller and globose to subglobose basidiospores.

**Holotype**: China. Yunnan Province: Yulong Naxi Autonomous County, 26°59′48″ N, 100°12′02″ E, altitude 2,880 m, in a coniferous and broad-leaved mixed forest with trees of Pinaceae and Fagaceae, 5 August 2019, Jian-Wei Liu 1,696 (KUN-HKAS129615).

**Description**:

- **Basidioma**: Very small.
- **Pileus**: 0.8–2.5 cm in diameter; convex to applanate, slightly depressed at the center, and sometimes slightly uplifted at the margin when mature; brownish orange (6A5–7) to brown (6E6–8) in color, sometimes flesh-colored (6A3–6) to brownish (6D4–7) at the margin; glabrous or sometimes with light white (1A1) pruinae; striate; context brownish orange (6A5–7) to brownish (6D4–7).
- **Lamellae**: Sinuate to adnate; distant; brownish orange (6A5–7) to brownish (6D4–7) in color; lamellulae attenuate.
- **Stipe**: 1.5–3 × 0.2–0.5 cm, cylindrical, hollow, brownish orange (6C7) to brown (6E6–8), with light white pruinae; basal mycelium white (1A1).
- **Pileipellis**: Composed of ± radially arranged, thick-walled (ca. 1 μm), orange-yellow filamentous hyphae 5–23 μm wide.
- **Basidia**: 43–53 × 10–14 μm, clavate, hyaline, 2-spored; sterigmata 7–14 μm long.
- **Basidiospores**: (excluding ornamentation) [60/3/3] 9–11 (−12) × (8.5–) 9–10.5 (−11.5) μm, $\bar{x}$ = 9.8 ± 0.6 × 9.5 ± 0.5, *Q* = 1–1.06 (−1.11), *Q*_m_ = 1.03 ± 0.03, globose to subglobose, hyaline, echinate, crowded; spines 0.5–2 μm long, up to 1.5 μm wide at the base; hilar appendix ca. 2 μm long, prominent, subtruncate.
- **Pleurocystidia and cheilocystidia**: Lacking.
- **Subhymenium**: Composed of filamentous hyphae that are 2–11 μm wide.
- **Lamellar trama**: Subregular, composed of thick-walled (ca. 0.5 μm), pale yellow filamentous hyphae that are 6–23 μm wide.
- **Stipitipellis**: Composed of appressed, parallel, simply septate, thick-walled (ca. 0.5 μm), colorless to pale grey hyphae that are 3–9 μm wide.
- **Caulocystidia**: Lacking.
- **Clamps**: Prominent and present in all parts of the basidioma.

**Habitat and distribution**: Found singly, scattered, or in groups on soil in subtropical broad-leaved forests with trees of the Fagaceae family. Basidioma occurs from summer to autumn. Known from southwest China (Yunnan).

Additional specimens studied: China:

Yunnan Province:

- Jingdong Yi Autonomous County, 24°27′02″ N, 100°12′46″ E, altitude 1,300 m, in a coniferous and broad-leaved mixed forest with trees of Pinaceae and Fagaceae, 30 July 2015, Qi Zhao 2570 (KUN-HKAS90444).
- Panlong District, 25°08′37″ N, 102°44′33″ E, altitude 1,953 m, in a coniferous and broad-leaved mixed forest with trees of Pinaceae and Fagaceae, 2 July 2021, Zhu-Liang Yang 6500 (KUN-HKAS122272).

## 4 Discussion

Only a few morphological characters of *Laccaria* species are available for taxonomy, and the color of the fruiting body varies widely (Mueller, 1992). Therefore, the application of molecular methods is necessary for the classification and identification of the *Laccaria species*. To date, the discovery of new species in *Laccaria* is rapidly increasing through molecular analyses (Popa et al., 2014, 2016; Vincenot et al., 2017; Cho et al., 2018; Cui et al., 2021), especially the multi-loci phylogenetic analysis (Sheedy et al., 2013; Wilson et al., 2017; Cho et al., 2020; Zhang et al., 2023). In this study, we determined three species as new through both molecular phylogenetic data and morphological features.

*Laccaria cinnabarina* is characterized by orange-red-colored basidiocarps with a conspicuously pellucid-striate pattern, fibrillose stipe, globose to subglobose, and echinulate basidiospores. According to our phylogenetic analysis, this species is related to *L. bicolor* and *L. trichodermophora*. However, *L. bicolor* can be distinguished from *L. cinnabarina* by its pinkish, fresh-colored, and non-striate pileus (Mueller, 1992). *L. trichodermophora* has a brownish orange non-striate and strongly pruinose to fibrillose pileus, subglobose to broadly ellipsoid basidiospores (Mueller, 1992).

On the other hand, *L. cinnabarina* is morphologically similar to *L. himalayensis*, *L. acanthospora*, *Laccaria aurantia* Popa et al., *L. fengkaiensis*, *Laccaria nanlingensis* Ming Zhang, and *L. umblilicata*, which are characterized by an orange pileus with striations. However, *L. himalayensis* differs by having characteristics such as large to very large basidioma, pastel red to grayish red-brown at the disk to orange-pink toward the margin of the pileus, globose basidiospores, and growing in mixed temperate alpine conifer forests (Wilson et al., 2013). *L. acanthospora* differs from *L. cinnabarina* by its orange, pink stipe, obellipsoid to globose basidiospores, and growing on sandy banks in mixed temperate alpine forests (Wilson et al., 2013).

*L. aurantia* differs from *L. cinnabarina* by its vividly orange pileus, stipe with a brownish-colored covering, and larger basidiospores (9–10 × 8–10 μm) (Popa et al., 2014). *Laccaria fengkaiensis* differs by its medium-sized to large basidioma, pastel red to grayish red lamellae, and smaller globose to obellipsoid basidiospores (5.2–6.3 × 5.1–6.3 μm) (Li, 2020). *Laccaria nanlingensis* differs by its pale red to grayish red lamellae, smaller basidiospores (6.5–7.5 × 6–7 μm), shorter spines (ca. 0.5–1 μm), and the presence of caulocystidia (Zhang et al., 2023). *Laccaria umblilicata* is characterized by its pale orange to light orange pileus, shorter, yellowish-white to orange-white stipe, and the presence of caulocystidia (Zhang et al., 2023).

*Laccaria longistriata* has a very small to small, brown to fresh-colored basidioma, a conspicuously striate to sulcate pileus, and globose to subglobose, echinate basidiospores (6.5–8 × 6–8 μm, *Q* = 1–1.07, *Q*_m_ = 1.02 ± 0.04) with spines that are 0.5–2 μm long and 0.5 μm wide at the base. Our phylogenetic analysis revealed that *L. longistriata* is related to *L. yunnanensis*, *L. vinaceoavellanea*, *L. pallidus*, *L. neovinaceoavellanea*, *L. fengkaiensis*, *L. rufobrunnea*, *L. lateritia*, *L. umblilicata*, and *L. prava*.

Indeed, *L. yunnanensis*, *L. vinaceoavellanea*, *L. pallidus*, *L. rufobrunnea*, and *L. prava* all have reddish to brownish basidiomata with prominent striate to sulcate pilei (Li, 2020; Zhang et al., 2023; Thapa et al., 2024). However, *L. yunnanensis* can be distinguished from *L. longistriata* by its medium-sized to large basidioma (6–10 diam.), slightly larger basidiospores (8–9 × 8–10 μm), and its occurrence in the Ailao Shan Mountains in Yunnan at approximately 2,500 m altitude, which is associated with the mid-montane humid evergreen broad-leaved forest (Popa et al., 2014). *Laccaria vinaceoavellanea* has a brownish vinaceous pileus, globose to subglobose basidiospores with relatively longer spines, which are 1.4–2.8 μm long (Hongo, 1971; Mueller, 1992; Wang et al., 2004). *Laccaria pallidus* is characterized by its medium-sized basidiocarps, globose, and smaller basidiospores (5.88–6.17 × 5.88–6.17 μm) (Thapa et al., 2024). *Laccaria rufobrunnea* is distinctive with its brownish orange to brownish red pileus and white to pinkish-white stipe (Zhang et al., 2023). *Laccaria lateritia* may be separated from *L. longistriata* by its 2-spored basidia (Popa et al., 2016). *Laccaria prava* differs from *L. longistriata* by its medium-sized to large basidioma, an orange stipe covered with brownish squamules, and basidiospores (7–8 × 6.5–7.5 μm) with relatively shorter spines, which are 0.5–1 μm long (Li, 2020). In addition, *Laccaria prava* is currently only known from Heishiding at approximately 100 m altitude, which is dominated by the monsoon evergreen broad-leaved forest in the south subtropical area of Guangdong Province (Li, 2020). Although *L. fengkaiensis* and *L. umblilicata* may be close to *L. longistriata*, these two species share orange-colored basidiomata (Li, 2020; Zhang et al., 2023). *Laccaria neovinaceoavellanea* is a unique species with a pastel pink to pale violet pileus in the lineage (Zhang et al., 2023).

Morphologically, *Laccaria rubroalba* Luo et al. and *Laccaria fulvogrisea* Popa et al. can be confused with *L. longistriata* due to their strongly striate to sulcate pileus and associations with broad-leaved trees (Popa et al., 2014; Luo et al., 2016). However, *L. rubroalba* has medium-sized, globose and subglobose and broadly ellipsoid basidiospores (6–9 × 5–7 μm) (Luo et al., 2016). *L. fulvogrisea* can be characterized by its grey to brownish basidioma with violet tinges and echinulate basidiospores with longer spines (1.7–2.5 μm long) (Popa et al., 2014). In addition, *Laccaria longistriata* also resembles *Laccaria striatula* (Peck) Peck (Bon, 1983), but the latter has a larger basidioma, has echinulate basidiospores with longer spines (1.4–2.8 μm long), and is abundant in wet mossy areas in eastern North America (Mueller, 1992; Popa et al., 2016).

*Laccaria spinulosa* is characterized by a brownish-orange basidiocarp and stipe with light white pruinae and globose to subglobose basidiospores. Our phylogenetic analysis revealed that specimens identified as *L. spinulosa* form a monophyletic lineage close to *L. acanthospora*, *L. canaliculata*, *L. galerinoides*, *L. miniata*, *L. glabripes*, *L. ohiensis*, and *L. paraphysata* and could be distinguished from the other known Asian *Laccaria* species that are currently recognized (Figure 1). *Laccaria acanthospora* differs from *L. spinulosa* by its small to large basidiocarps, orange pileus, obellipsoid to globose basidiospores, and its occurrence on sandy banks in mixed temperate alpine forests (Wilson et al., 2013).

*Laccaria canaliculate* differs from its submembranous, velvety, light brown pileus, which is slightly crenulate, and a pallid stipe (Mueller, 1992). *Laccaria galerinoides* differs by having a light ochraceous-brown to golden-ochraceous pileus, a longer and tubular stipe, and ellipsoid basidiospores (Mueller, 1992). *Laccaria miniata* differs by its red pileus, subglabrous to fibrillose stipe, and occurrence in north-subtropical habitats (Zhang et al., 2023). All three of the latter species have a reddish-brown pileus. However, *L. ohiensis*, originally reported from North America, differs by having a glabrous to finely fibrillose stipe with a striate to slightly longitudinally striate and smaller basidiospores (7.7–9.4 × 7–9 μm) (McNabb, 1972). The other two species were originally reported from New Zealand.

*Laccaria spinulosa* can be distinguished from other small, brownish-orange, plicate-striate *Laccaria* taxa, such as *L. fagacicola* Y. Y. Cui et al., *L. araneosa* H. J. Cho and Y. W. Lim, *L. parva* H. J. Cho and Y. W. Lim, *L. torosa* H. J. Cho and Y. W. Lim, *L. rufobrunnea*, by its 2-sterigmate basidia that bear large basidiospores with long and broad echinulae (Cho et al., 2018; Cui et al., 2021).

In addition, the macroscopic features of *L. spinulosa* are similar to those of *Laccaria* species with two-spored basidia, namely *L. pumila*, *Laccaria tortilis* (Bolton) Cooke, and *Laccaria nigra* Hongo. However, *L. spinulosa* can be differentiated from these three taxa in the following ways:

- **Basidiospores**: *Laccaria spinulosa* has a relatively smaller diameter (9–11 × 9–10.5 μm) and globose to subglobose basidiospores compared to *L. pumila* and *L. tortilis* (Mueller, 1992).
- **Pileus**: *L. nigra* has a grayish pileus and globose basidiospores (Mueller, 1992), whereas *L. spinulosa* does not.

It is essential to include more *Laccaria* species from different regions of China, combining morphological characters, molecular data, and ecological information, to understand the species diversity of *Laccaria* in China in future studies. In terms of morphology, the size of basidia, the presence of two- or four-spored basidia, and the length and density of spines on the spore surface can provide useful directions for species identification.

## Data availability statement

The datasets presented in this study can be found in online repositories. The names of the repository/repositories and accession number (s) can be found in the article/supplementary material.

## Author contributions

JL: Writing – original draft, Conceptualization, Software. N-JC: Formal analysis, Writing – original draft. Y-YC: Writing – review & editing, Conceptualization, Methodology.
